# Editorial: Cytokines and Intestinal Mucosal Immunity

**DOI:** 10.3389/fimmu.2021.698693

**Published:** 2021-05-14

**Authors:** Theresa T. Pizarro, Charles A. Dinarello, Fabio Cominelli

**Affiliations:** ^1^ Department of Pathology, Case Western Reserve University School of Medicine, Cleveland, OH, United States; ^2^ Department of Medicine, University of Colorado Denver, Aurora, CO, United States; ^3^ Digestive Health Research Institute, Case Western Reserve University School of Medicine, Cleveland, OH, United States

**Keywords:** cytokines, gut mucosal immunity, innate immunity, adaptive immunity, intestinal epithelium, inflammatory bowel disease

Since discovery of the prototypic cytokines, interleukin-1 (IL-1) and tumor necrosis factor-α (TNF), almost 50 years ago (1, 2), an explosion of information has followed regarding the biology of cytokines and their critical role(s) during health and disease. To date, 41 interleukins and more than 18 TNF superfamily (TNFSF) members have been described. Notably, in 1990, our group was one of the first to show that blockade of a single cytokine, *i.e*., IL-1, was effective in markedly reducing the severity of experimental colitis (3), laying the foundation to conceptualize that targeting of an individual cytokine could successfully impact the development and progression of a specific disease. The role of cytokines, in fact, has been particularly important in the gastrointestinal tract, both in maintaining homeostasis and during chronic inflammatory disorders, such as inflammatory bowel disease (IBD), wherein many cell types have the ability to both react to, and produce, cytokines in response to a variety of antigenic stimuli, dietary products, microbial components, and toxic agents. This wealth of new information has led to the approval of different anti-cytokine therapies, such as anti-TNF and anti-IL-12/23 monoclonal antibodies, for the treatment of both Crohn’s disease (CD) and ulcerative colitis (UC), the two main forms of IBD. In addition, novel small molecule inhibitors, such as those targeting the JAK/STAT pathway, and which possess broad anti-cytokine activity, are now available in the armamentarium of gastroenterologists to treat IBD.

In this Research Topic, the role of canonical, and more novel, cytokines are discussed in the context of intestinal immunity and chronic gut inflammation. Three articles focus on the role(s) of TNFSF members (Li et al.; Valatas et al.; Giles et al.). Specifically, Li et al. and Valatas et al. report the importance of the TL1A (TNF-like ligand 1A, TNFSF15)/DR3 (death receptor 3,TNFRSF25) ligand-pair, for which increasing evidence suggests a critical role not only in the pathogenesis of IBD, but also in the development of gut fibrosis/fibrostenotic disease. These papers highlight TL1A/DR3’s pleiotropic functions in regulating the balance between T effector and T regulatory cells (Tregs), as well as innate lymphoid cells (ILCs), thereby serving as a vital rheostat during IBD. Notably, monoclonal antibodies against TL1A are currently in clinical trials for the treatment of CD and UC, and will shortly reveal the efficacy of anti-TL1A/DR3 strategies in IBD. Additionally, Giles et al. show that LIGHT (TNFSF14) can have differential effects on the outcome of experimental colitis, depending on engagement of, and signaling through, its various cognate receptors, further emphasizing the complexity of the TNFSF and the function of its members in IBD.

Also included in the Research Topic are comprehensive reviews highlighting the contribution of key populations of cytokine-producing, and -responding, gut mucosal cells(Andrews et al.; De Salvo et al.; Weidinger et al.). Intestinal epithelial cells play a central role in maintaining homeostasis throughout the gastrointestinal tract as collectively, they represents the host’s first line of defense against the external environmental, while balancing their response to the underlying gut mucosal immune system. Dysregulation of these finely-tuned interactions can compromise barrier function and result in uncontrolled, chronic inflammation. Andrews et al. provide important information regarding how epithelial-derived cytokines, as well as cytokines affecting the gut epithelium, orchestrate crucial functions during health and disease states. Furthermore, ILCs are a relatively new family of heterogenous immune cells driven by specific transcription factors that exhibit distinct cytokine profiles and are particularly enriched at mucosal barriers. As their name implies, ILCs provide necessary innate immunity to the host; however, based on their strategic location, they must also be able to temper and quickly alter response(s) to their dynamic and everchanging surroundings. In this context, De Salvo et al. summarize what is currently known regarding the cytokines that enable ILC plasticity, and their ability to readily transdifferentiate, leading to either protective or pathogenic functions within the gut. Interestingly, the original article from Christodoulou-Vafeiadou et al. underscores the important contributions of epithelial versus innate immune cells, reporting that the RNA binding protein, HuR, that post-transcriptionally regulates mRNAs encoding, among others, cytokines, confers both convergent as well as divergent functions in the gut depending on its cellular source and what type of challenge is presented to the host (*e.g*., colitis-associated cancer vs. infectious colitis). Finally, a newly emerging area of interest in cytokine biology is the role adipokines, which are soluble factors released by fat tissue. Their role in IBD is especially relevant since CD is often characterized by hyperplasia of the mesenteric fat situated around affected, inflamed segments of the small intestine, and notably, this so-called ‘creeping fat’ has also been implicated in fibrostenotic CD, with recent work demonstrating its regulation by live bacteria derived from the gut microbiome (4). Weidinger et al. provide important information regarding adipokines, its regulation of creeping fat, and put forth a working model as to how these factors participate in gut health and disease.

The remaining contributions focus on various aspects of targeting cytokines to treat IBD. Anti-α4β7 integrin therapy using vedolizumab (VDZ) was introduced a decade ago as an effective, alternative option for IBD patients that were refractory to either conventional treatments or TNF inhibitors. Ironically, Rath et al. show that increased activation of TNF is observed in IBD patients unresponsive to VDZ, and that anti-TNF therapy may actually be more effective in VDZ refractory patients. In regards to newer anti-cytokine strategies, blocking the IL-12/IL-23 axis has proven to be effective for both CD and UC patients. Interestingly, although Th17 cells are known to expand in response to IL-23, anti-IL-17 treatment is reported to be ineffective in treating CD, and in fact, worsens disease (5). In support of disease specificity for anti-cytokine therapy, Buchele et al. show that blockade of the IL-23/Th17 pathway is ineffective in preventing colitis in a mouse model of graft-vs.-host-disease, suggesting that targeted anti-cytokine therapy is disease specific, rather than organ-specific, and that the underlying etiology is critical to consider. Regulation of another cytokine recently tested in CD is TGFβ1, whose anti-inflammatory activity is restored by administering Mongersen, an oral SMAD7 antisense oligonucleotide, but with controversial outcomes. Troncone et al. review this important cytokine pathway in intestinal immunity that, despite negative results of Phase III clinical trials (6), remains an interesting therapeutic target for IBD, perhaps using a different drug formulation. In closing, Abo et al. contribute an original article demonstrating the efficacy of combination IL-2 immunocomplex and anti-IL-5 in experimental colitis through a mechanism that involves expansion of Foxp3^+^ Tregs. Although premature to consider for IBD treatment, the concept of combination therapy is certainly an attractive strategy, particularly for complex, multi-pathway disease processes that are prevalent in disorders like IBD. We anticipate that the field of cytokines in intestinal immunity will continue to grow and evolve, which will lead to a better understanding of pathogenic mechanism(s) and discovery of novel anti-cytokine treatments for chronic gut inflammation.

## Author Contributions

TTP, FC and CAD contributed equally to conceptualizing, writing, and editing the final submitted manuscript. CAD contributed to editing the final submitted manuscript. All authors contributed to the article and approved the submitted version.

## Conflict of Interest

The authors declare that the research was conducted in the absence of any commercial or financial relationships that could be construed as a potential conflict of interest.

## Abbreviations

CD, Crohn’s disease; DR3, death receptor 3; IL-, interleukin-; ILC, innate lymphoid cell; TGFβ1, transforming growth factor-beta1; Th, T-helper; TL1A, TNF-like ligand 1A; TNF, tumor necrosis factor-α; TNFSF, TNF super family; Treg, T-regulatory cell; UC, ulcerative colitis; VDZ, vedolizumab.

## References

[1] Carswell EA Old LJ Kassel RL Green S Fiore N Williamson E. An Endotoxin-Induced Serum Factor That Causes Necrosis of Tumors. Proc Natl Acad Sci USA (1975) 72(9):3666–70. 10.1073/pnas.72.9.3666 PMC433057 1103152

[2] Dinarello CA Renfer L Wolff SM. Human Leukocytic Pyrogen: Purification and Development of a Radioimmunoassay. Proc Natl Acad Sci USA (1977) 74(10):4624–7. 10.1073/pnas.74.10.4624 PMC431999 22079

[3] Cominelli F Nast CC Clark BD Schindler R Lierena R Eysselein VE. Interleukin-1 (IL-1) Gene Expression, Synthesis, and Effect of Specific IL-1 Receptor Blockade in Rabbit Immune Complex Colitis. J Clin Invest (1990) 86(3):972–80. 10.1172/JCI114799 PMC296817 2168444

[4] Ha CWY Martin A Sepich-Poore GD Shi B Wang Y Gouin K. Translocation of Viable Gut Microbiota to Mesenteric Adipose Drives Formation of Creeping Fat in Humans. Cell (2020) 183(3):666–83. 10.1016/j.cell.2020.09.009 PMC7521382 32991841

[5] Hueber W Sands BE Lewitzky S Vandemeulebroecke M Reinisch W Higgins PDR. Secukinumab, a Human anti-IL-17A Monoclonal Antibody, for Moderate to Severe Crohn’s Disease: Unexpected Results of a Randomised, Double-Blind Placebo-Controlled Trial. Gut (2012) 61(12):1693–700. 10.1136/gutjnl-2011-301668 PMC4902107 22595313

[6] Sands BE Feagan BG Sandborn WJ Schreiber S Peyrin-Biroulet L Colombel JF. Mongersen (GED-0301) for Active Crohn’s Disease: Results of a Phase 3 Study. Am J Gastroenterol (2020) 115(5):738–45. 10.14309/ajg.0000000000000493 31850931

